# The benefits of data mining

**DOI:** 10.7554/eLife.30280

**Published:** 2017-08-16

**Authors:** Audrey Bone, Keith Houck

**Affiliations:** National Center for Computational Toxicology, Office of Research and DevelopmentUS Environmental Protection AgencyResearch Triangle ParkUnited States

**Keywords:** adverse drug reactions, FAERS, big data, data analysis, Human

## Abstract

Careful analysis of a database populated by physicians and patients sheds new light on the side effects of drugs.

**Related research article** Maciejewski M, Lounkine E, Whitebread S, Farmer P, DuMouchel W, Shoichet BK, Urban L. 2017. Reverse translation of adverse event reports paves the way for de-risking preclinical off-targets. *eLife* **6**:e25818. doi: 10.7554/eLife.25818

The goal of pharmaceutical drug development is to produce compounds that can treat medical conditions effectively without causing side effects (which are known as adverse drug reactions in the pharmaceutical industry). It has been estimated that serious versions of adverse drug reactions occur in over two million patients per year in the US, with 100,000 of them resulting in deaths (Giacomini et al., 2007). Potential new drugs are subject to in vivo testing with laboratory animals and in vitro studies in cell lines before they are ever used in human clinical trials. However, humans differ from laboratory animals in many ways and there are limitations to the applicability of in vitro studies. Therefore, adverse drug reactions (ADRs) are often not identified until a drug is tested in a clinical trial, which can result in costly failures.

Moreover, even if a drug is approved for use after clinical trials, some critical ADRs only become apparent after a large number of patients have been treated over a long time. This is because it can be difficult to account for a number of important factors in clinical trials, such as patient age, co-exposures to other drugs, genetic differences, environmental and dietary variances, and long-term use (Woodcock, 2016). The diet drug fenfluramine-phentermine (fenphen), for example, had to be withdrawn in 1997 following patient deaths that resulted from a drug metabolite binding to an off-target receptor (5HT_2B_) that caused a heart valve disease (Rothman et al., 2000). Hence, there is a real need for methods that can predict ADRs much earlier in the drug development process.

Current methods to predict ADRs are limited. Many animal models do not adequately predict human responses (Kullak-Ublick et al., 2017; FDA, 2017), and while in vitro studies can examine the molecular pathways underlying an adverse reaction, we need to know something about the mechanisms driving the ADR in the first place. The receptor implicated in fenphen toxicity is an example of a molecular target that compounds can be tested against with in vitro assays, as is an ion-channel protein called hERG that has been linked to heart arrhythmias (Roy et al., 1996). However, the majority of ADRs do not have known underlying mechanisms.

Now, in eLife, Mateusz Maciejewski of Pfizer, Brian Shoichet of UCSF, Laszlo Urban of Novartis and colleagues report a third approach that involves analyzing a large, crowd-sourced database of ADRs maintained by the Food and Drug Administration (FDA) in the United States (Maciejewski et al., 2017). The FDA Adverse Event Reporting System (FAERS) is a publicly accessible and voluntary database that allows physicians, pharmacists and patients (and also lawyers involved in drug litigation) to report adverse events associated with prescription or over-the-counter medicines, along with nutritional products, cosmetics and food/beverages (Sakaeda et al., 2013). The database now contains over nine million records reaching back to 1969 and continues to grow rapidly (Figure 1). However, while FAERS contains a wealth of real world information, these data must be handled with care.

**Figure 1.**
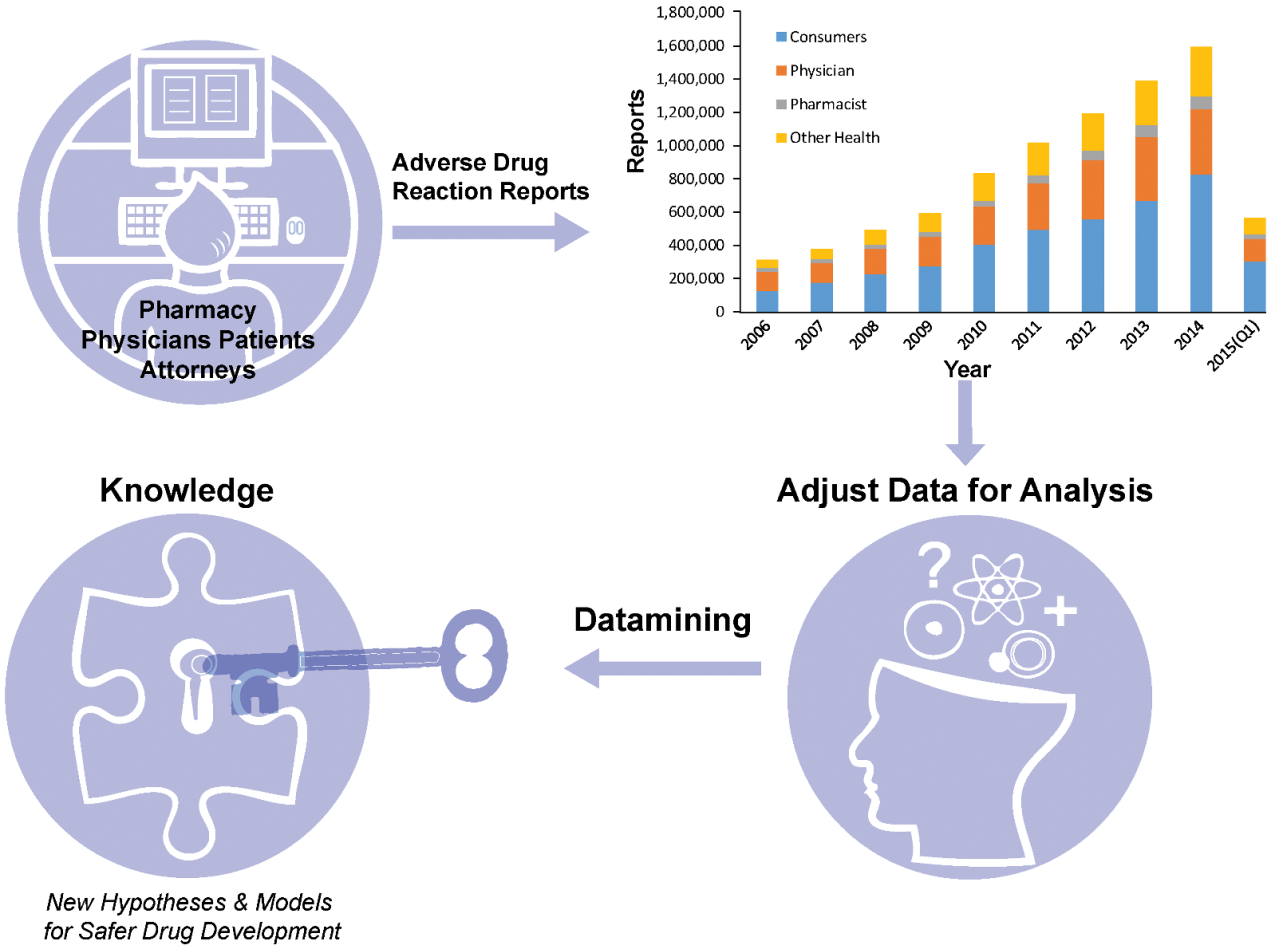
Making the most of the FDA Adverse Event Reporting System (FAERS). Patients, physicians, pharmacists and other health-care professionals input information about adverse drug reactions into the FAERS database. Maciejewski et al. have shown that it is possible to use data mining and statistical analysis to extract new insights about adverse drug reactions from the database: the first step is to deal with the noise and other problems associated with such crowd-sourced databases. The amount of reports in FAERS has grown rapidly over the past decade (top right; data from FDA).

As an example, FAERS uses names rather than chemical structures to identify drugs, with each chemical structure having an average of 16 different names (or 378 in the case of fluoxetine, also known as Prozac), so Maciejewski et al. were required to first aggregate all the information associated with each chemical structure. They also had to remove redundant data (e.g., where the same event was entered multiple times) and other data that were misleading (e.g., when the adverse event was actually a pre-existing medical condition).

Using various data visualization techniques, Maciejewski et al. were then able to begin to dig deeper into the data and identify a number of potentially confounding factors that may impact overly simplistic interpretations. Data for individual drugs plotted chronologically showed distinct spikes in reports that could be tied to specific events. For example, initial reports of cardiovascular and cerebrovascular events associated with rofecoxib (the nonsteroidal anti-inflammatory drug with the brand name Vioxx) resulted primarily from physician reports. The number of reports later increased dramatically, first due to patients and later due to lawyers, following the publication of a clinical study linking Vioxx to cardiovascular events and, two years later, when warnings were added to the Vioxx label.

An analysis of the diabetes drugs rosiglitazone and pioglitazone (which have similar structures) illustrated how the database can be used to differentiate between a class effect (in which an effect is seen across an entire class of drugs) and a drug-specific effect. Rosiglitazone showed a strong signal of cardiovascular events, such as congestive cardiac failure, that persisted over time. Pioglitazone, on the other hand, showed only a small, inconsistent spike in cardiovascular events that coincided with the increased public scrutiny of rosiglitazone. Over time, a strong bladder cancer signal appeared for pioglitazone that was not seen with rosiglitazone. This result is supported by recent epidemiological studies which suggest that differences in receptor selectivity are responsible for the differences between the drugs (Tuccori et al., 2016).

Maciejewski et al. also illustrated the need to take pharmacokinetics into account when analyzing the FAERS database by examining hypertension associated with a cancer treatment involving the inhibition of vascular endothelial growth factor receptor. Nineteen different inhibitors were analyzed and only those with exposure margins (the ratio of the potency against the target to the patient’s serum concentration) under 10 were linked to hypertension. Maciejewski et al. concluded that this could be used as a drug development guideline for this class of compounds and that the use of exposure margins in the FAERS analysis may help define drugs that cause adverse events.

Crowd-sourced databases are often noisy and subject to interference from many factors because the data are entered by non-experts. However, such databases can be an invaluable resource when analyzed appropriately. Maciejewski et al. have shown how to handle the noise in the FAERS database and the limitations of the database structure, and how to deal with social factors such as news reports, drug recalls, and ongoing litigation. Moreover, using relatively simple statistical methods, they demonstrated how to extract useful information about adverse events (including information about relationships and mechanisms) from the data. Their work will also provide a foundation for the use of sophisticated methods (such as empirical Bayesian statistics and hierarchical methods) in future studies. The recommendations they make for improving the database, such as including pharmacokinetics information, would make it even more valuable.

## Note

The views expressed in this paper are those of the authors and do not necessarily represent the views or policies of the US Environmental Protection Agency.
